# Protective Effects and Potential Mechanisms of D-Aspartate on Testicular Damage Induced by Polystyrene Microplastics

**DOI:** 10.3390/biom15111484

**Published:** 2025-10-22

**Authors:** Sara Falvo, Giulia Grillo, Imed Messaoudi, Nada Fradi, Gabriella Chieffi Baccari, Maria Maddalena Di Fiore, Alessandra Biasi, Maria Rosaria Ambruosi, Alessandra Santillo, Massimo Venditti

**Affiliations:** 1 Dipartimento di Scienze e Tecnologie Ambientali, Biologiche e Farmaceutiche, Università degli Studi della Campania ‘Luigi Vanvitelli’, Via Vivaldi, 43, 81100 Caserta, Italy; sara.falvo@unicampania.it (S.F.); giulia.grillo@unicampania.it (G.G.); alessandra.santillo@unicampania.it (A.S.); 2 LR11ES41: Génétique, Biodiversité et Valorisation des Bioressources, Institut Supérieur de Biotechnologie, Université de Monastir, Monastir 5000, Tunisia; imed_messaoudi@yahoo.fr (I.M.); fradinada123@gmail.com (N.F.); 3 Dipartimento di Medicina Sperimentale, Sez. Fisiologia Umana e Funzioni Biologiche Integrate, Università degli Studi della Campania ‘Luigi Vanvitelli’, Via Santa Maria di Costantinopoli, 16, 80138 Napoli, Italy; alessandra.biasi@unicampania.it (A.B.); mariarosaria.ambruosi@unicampania.it (M.R.A.); massimo.venditti@unicampania.it (M.V.)

**Keywords:** Polystyrene Microplastics, D-aspartate, reproduction, oxidative stress, apoptosis, autophagy, steroidogenesis, spermatogenesis

## Abstract

Polystyrene Microplastics (PS-MPs) affect testicular activity, as evidenced by increased oxidative stress, apoptosis, and autophagy activation, impairing steroidogenesis and spermatogenesis. The present study investigates, for the first time in vivo, the potential protective effect of D-aspartate (D-Asp) against PS-MPs-induced damage on the testicular function of adult rats. D-Asp, well-known stimulator of testosterone biosynthesis and spermatogenesis progression, possesses pharmacological properties, including antioxidant and anti-apoptotic ones. The results showed that PS-MP’s adverse effects on testicular activity were reversed by D-Asp treatment. Mechanistically, D-Asp inhibited testicular oxidative stress by modulating the protein levels of CAT, SOD1, SOD2, and 4-HNE; affecting TBARS levels; and reducing apoptosis, as suggested by CYT C analysis and a TUNEL assay. Furthermore, D-Asp administration mitigated PS-MPs-induced autophagy activation by modulating the expression of LC3BI, LC3BII, and p62 proteins. Finally, the amino acid counteracts PS-MPs damage on steroidogenesis and spermatogenesis by restoring normal levels of steroidogenic (StAR, 3β-HSD, and 17β-HSD) and spermatogenic (PCNA and SYCP3) markers. This study encourages further research to understand the potential value of the amino acid in improving human testicular health and male fertility.

## 1. Introduction

Male reproductive health and fertility rates have declined over the past 30 years. This trend in modern society has been attributed to routine exposure to toxins and xenobiotics through food, drink, and environmental pollution [1,2,3]. Many studies have demonstrated that the testis is a primary target of environmental contaminants, the accumulation of which leads to testicular dysfunction, resulting in reduced gamete quality [4,5,6].

MPs are a focus of attention among environmental pollutants, as all organisms are constantly exposed to them [7,8]. Indeed, considering the overuse and durable nature of plastics, the spread of MPs in the environment and their impact on human health is inevitable. MPs are particles less than 5 mm in diameter derived from any plastic through various physical, chemical, and biological processes [9,10]. Ubiquitous in all environments—aquatic, terrestrial, and atmospheric [11,12,13]—they easily reach all organisms, including humans, through the food chain but also polluted water and inhalation of polluted air [14,15].

Recently, PS-MPs have been demonstrated to penetrate testicular tissue in mice [16] and rat models [17,18,19]. MP exposure can cause damage to sperm in rodents, resulting from the abnormal differentiation of mature gametes [18,20,21,22]. MPs-induced defects in spermatogenesis are due to increased inflammation, oxidative stress, apoptosis, and activation of autophagy in the testicular tissue [17,18,20,21,22,23]. On the other hand, PS-MPs also induce testicular dysfunction by impairing rat LC, resulting in decreased serum T levels [19,24]. Consistently, it has been demonstrated in vitro that PS-MPs accumulate in mouse LC and reduce the mRNA [25] and protein expression levels of key steroidogenesis enzymes (StAR, 3β-HSD, and 17β-HSD) [26].

Therefore, it is becoming increasingly important to identify molecules that can act as protective agents against reprotoxicity induced by environmental pollutants. Moreover, nowadays, most people leading a healthy lifestyle tend to use natural products such as supplements, nutraceuticals, complementary medicine, or alternative treatments. In this context, D-aspartate (D-Asp), an endogenous amino acid present in the testes, reduced the testicular oxidative status. Specifically, in vivo administration of D-Asp to adult rats significantly reduced the level of TBARS, a byproduct of lipid peroxidation, and stimulated the enzymatic activity/protein expression of the antioxidant enzymes superoxide dismutase (SOD) and catalase (CAT) [27,28]. Indeed, by mediating the activity of antioxidant enzymes, D-Asp exerts a notable anti-apoptotic effect in the testis [29]. Still, more importantly, this amino acid is known to play a fundamental role in male fertility by promoting testosterone (T) biosynthesis and upregulating androgen receptor expression [30,31,32,33,34]. Furthermore, in vitro studies have revealed the promising effects of D-Asp on germ cell (GC) proliferation [35,36,37] and the improvement in sperm quality [38,39,40,41]. Therefore, due to the multiple roles played by D-Asp in male reproductive health, pharmaceutical preparations containing the amino acid have been developed and are currently used in cases of male infertility [34,35,36,37,38].

Our research is currently focused on investigating the potential role of D-Asp in counteracting or mitigating the damage induced by environmental pollutants on testicular function. In this regard, we recently demonstrated that D-Asp can counteract/prevent the damage induced by cadmium [29] and ethane dimethane sulfonate [42] in rat testes.

To date, while the deleterious effects of PS-MPs on male reproductive function have been documented well, no in vivo studies have investigated the potential protective role of D-Asp against this specific environmental insult. Therefore, relying on D-Asp’s potential, we evaluated its possible action in alleviating the adverse effects induced by PS-MPs treatment on rat testis.

## 2. Materials and Methods

### 2.1. PS-MPs Particles

In this study, pristine PS-MPs (PS/Q-R-KM491; GmbH, Berlin, Germany) were employed for experiments [43]. They were spherical particles with a diameter of 5 μm. Particle size was measured with non-invasive backscatter optics. The ENM ζ-potential was also measured via Zetasizer Nano ZS (Malvern Panalytical Ltd., Malvern, Worcestershire, UK). Particle size was independently monitored by our partner in CNRS, ICMPE, University of Creteil, Paris, France. An in-house assessment of the polystyrene spheres revealed an average particle agglomerate size of 4.98 μm ± 0.15 and a ζ-potential of −0.0934 ± 0.172 [18].

### 2.2. Rats and Experimental Design

Thirty-five male Wistar rats *Rattus norvegicus* aged 60 days were maintained in single stainless-steel cages, provided with Aspen Bricks and Aspen Balls as environmental enrichment, under standard controlled conditions of light (12:12 light/dark schedule), temperature (22 ± 2 °C), and humidity (55 ± 20%). Since rats share approximately 75–80% of their genetic makeup with humans, they represent a suitable model for research into human diseases. The Ethics Committee approved the experimental protocol for Research in Life and Health Sciences of the Higher Institute of Biotechnology in Monastir (CER-SVS/ISBM- protocol 022/2020; approved date: 3 July 2023). It was conducted following the UNESCO Recommendation on Science and Scientific Research (1974, 2017). All animal experiments complied with ARRIVE guidelines. At the start of the study, and after seven days of acclimatization, the healthy rats were arbitrarily allocated into the cages and then randomly divided into seven groups (five animals each). The minimum sample size (n = 5) for the study was calculated using the ANOVA (fixed effects, omnibus, and one-way) method outlined in G*power software, version 3.1.9.7. An effect size (f) of 0.88 was assumed. With an alpha of 5% and power of 95%, the sample size was found to be 5 animals per group (7 experimental groups). Control groups (C 30 and C 45) received 2 mL of distilled water/day/rat via gastric gavage for 15 or 45 days; D-Asp group received 0.1 mM D-Asp/day/g body weight via gastric gavage for 15 days (D-Asp; #219096; Sigma-Aldrich, Milan, Italy); PS-MPs group received 0.1 mg of MPs day/g body weight via gastric gavage for 30 days; PS-MPs + D-Asp group was treated with MPs (in the morning) and then with D-Asp (in the afternoon) for 30 days; D-Asp/PS-MPs group was treated with D-Asp for 15 days and then with MPs for 30 days; PS-MPs/D-Asp group was treated with MPs for 30 days and then with D-Asp for 15 days. To minimize potential confounders, the cages were labeled with the name of the experiment group, respectively. The animals received a standard laboratory rodent diet and drinking water ad libitum. Rats were weighed every 5 days.

The MP solution was prepared as follows: l mg of PS-MPs was diluted in 5 mL of Millipore Milli-Q water and processed via ultrasonic vibration, and 0.5 mL of the resulting solution was given via oral gavage once daily (0.1 mg/day, which corresponds to 1.5 × 10^6^ particles/day).

The experimental design was chosen based on previous studies indicating that PS-MPs accumulate in the testes following daily administration of 0.1 mg of PS-MPs for 30 days via gastric gavage [18]. Similarly, numerous studies have shown that D-Asp accumulates in the testis after oral administration for 15 days [18,19,29,34,43].

Every effort was made to minimize animal pain and suffering. During the experiment, nutritional and hydration status, health, environmental and hygiene conditions, and behavioral status of the animals were monitored daily to identify any signs of pain, anxiety, or suffering so that appropriate measures could be taken.

All the rats survived well during the period of the experiment. No appreciable change was observed in their behavior, appetite, and motor activity during the experimental period. There were no exclusions of animals, experimental units, or data points for the determinations described below. The researchers who performed the different stages of the experiment were blinded to the group assignment.

### 2.3. Sample Collection

After treatments, all animals were anesthetized via intraperitoneal injection of 4% chloral hydrate (10 mL/Kg b.w.) and then euthanized via decapitation. The testes were removed and weighed. One testis was fixed in 10% neutral buffered formalin for histological and IF analysis, while the other was stored at −80 °C for biomolecular investigations.

The epididymes were dissected and minced in PBS (pH 7.4) to allow the release of SPZ from the ducts. The resulting fluid was filtered and examined under a light microscope to ensure the absence of contamination by other cell types. Next, aliquots were placed onto slides, air-dried, and stored at −20 °C. The remaining samples were centrifuged at 1000× *g* for 15 min at 4 °C and stored at −80 °C for molecular analysis.

### 2.4. Sperm Parameter Evaluation

All primary sperm parameters were assessed following established methodologies from previous studies [18]. For morphological evaluation, the sperm suspension obtained from the left epididymis (n = 5/group) was diluted in water to a final volume of 20 mL. Eosin (1%) was added (1–2 mL per 20 mL suspension) and incubated at room temperature for 1 h. A drop of the stained suspension was then placed on a slide, smeared, and examined under a light microscope (Axiostar Plus Zeiss, Carl Zeiss Microscopy GmbH, Jena, Germany) at 40× magnification. A total of 300 spermatozoa (SPZ) were analyzed per slide, and the percentage of abnormal SPZ was calculated.

For motility assessment, SPZ obtained from the right epididymis were diluted at a 1:10 ratio. A 20 µL aliquot of the diluted suspension was placed in a Malassez cell, and mobile and non-motile SPZ were counted using an optical microscope (Axiostar Plus Zeiss) at 40× magnification. A total of 300 SPZ were evaluated per slide, and motility was expressed as the percentage of motile SPZ relative to the total count.

### 2.5. Determination of T Levels

T levels were determined via the ELISA method using a commercial kit (#DKO002, DiaMetra, Milan, Italy) in the testes of C, D-Asp-, PS-MP-, PS-MP + D-Asp-, PS-MP/D-Asp-, and D-Asp/PS-MP-treated rats. Briefly, testes (n = 5/group) were homogenized 1:10 (*w*/*v*) with PBS 0.01 M, pH 7.5. The homogenate was then mixed with ethyl ether (1:10 *v*/*v*), and the ether phase was withdrawn after centrifugation at 3000× *g* for 10 min. The upper phase (ethyl ether) was transferred to a glass tube and evaporated on a hot plate at 40 °C to 50 °C under a hood. The residue was dissolved in PBS 0.01 M, pH 7.5, containing BSA 5%, and then used for the assay. The sensitivity assay for T was 32 pg/mL, and the intra-CV and the inter-CV were 13.7% and 7.5%, respectively.

### 2.6. Evaluation of TBARS Levels

Testis lysates (see point “Protein extraction”) (n = 5/group) were used to determine TBARS levels, as reported by Satoh, 1978 [44]. Results were expressed as TBARS μM/μg of extracted protein. Each measurement was performed in triplicate.

### 2.7. Protein Extraction and WB Analysis

Testis (n = 5/group) was homogenized directly in RIPA lysis buffer (#TCL131; Hi Media Laboratories GmbH; Einhausen, Germany) containing 10 μL/mL of protease inhibitors mix (#39102; SERVA Electrophoresis GmbH; Heidelberg, Germany), then centrifuged at 14,000× *g* for 20 min. Bradford assay (Bio-Rad, Inc., Hercules, CA, USA) was used to determine protein concentration. Fifty micrograms of total protein extracts was separated into SDS-PAGE (13% polyacrylamide) and treated as described by Venditti et al. (2023b) [29]. For details concerning all the used primary and secondary antibodies, see Table S1. The amount of proteins was quantified using ImageJ software (version 1.53 t; National Institutes of Health, Bethesda, MD, USA). Each WB was performed in triplicate.

### 2.8. Histology and IF Analysis

The fixed testes (n = 5/group) were dehydrated through an increasing percentage series of ethanol, cleared in xylene, and embedded in paraffin. Five-micrometer slices were obtained, stained with hematoxylin and eosin, and examined under a light microscope (Leica DM 2500, Leica Microsystems, Wetzlar, Germany). For histopathological evaluation, 30 seminiferous tubules/animal, for a total of 150 tubules per group, were counted, and photographs were taken using a Leica DFC320 R2 Digital Camera (Leica Microsystems, Wetzlar, Germany).

For IF analysis, the slides were then incubated with specific primary and secondary antibodies (Table S1). After incubation, they were mounted with Vectashield containing DAPI (#H-1200–10; Vector Laboratories, Peterborough, UK) for 5 min and covered with coverslips. The slides were observed under a fluorescent microscope with a UV lamp (Leica DM5000 B + CTR 5000; Leica Microsystems, Wetzlar, Germany) and saved using IM 1000 software (version 4.7.0; Leica Microsystems, Wetzlar, Germany). Photographs were taken using a Leica DFC320 R2 digital camera.

Densitometric analysis of IF signal intensity was conducted using the Fiji plugin (version 3.9.0/1.53t) of ImageJ software. A total of 30 tubules per animal were analyzed, corresponding to 150 tubules per group. Each IF experiment was performed in triplicate.

### 2.9. TUNEL Assay

Apoptotic cells were identified using the TUNEL assay with the DeadEnd™ Fluorometric TUNEL System (#G3250; Promega Corp., Madison, WI, USA), following the manufacturer’s instructions with few modifications. Briefly, before the incubation with reaction mix, sections of testis (n = 5/group) were blocked with 5% BSA (#A1391; AppliChem GmbH, Darmstadt, Germany) and normal goat serum (#S26-M; Sigma Aldrich; Milan, Italy), diluted 1:5 in PBS, and then treated with Peanut agglutinin (PNA) lectin to mark the acrosome. Nuclei were counterstained with Vectashield containing DAPI (#H-1200-10, Vector Laboratories, Inc., Newark, CA, USA). To evaluate the % of TUNEL-positive cells, 30 seminiferous tubules/animal for a total of 150 tubules per group were counted using the Fiji plugin (version 3.9.0/1.53 t) in ImageJ Software. TUNEL assay was performed in triplicate.

### 2.10. Statistical Analysis

Statistical analysis was performed using GraphPad Prism9 (GraphPad SoftwarLLC, San Diego, CA, USA), www.graphpad.com). To evaluate significant changes among experimental groups, a one-way analysis of variance (ANOVA) followed by a Student–Newman–Keuls test was used. Values for *p* < 0.05 were considered statistically significant. All data were expressed as the mean ± S.D.

## 3. Results

To investigate the putative role of D-Asp in counteracting/preventing testis damage induced by PS-MPs, in a group of rats, D-Asp was simultaneously administered with MPs (PS-MPs + D-Asp group), while to another group, D-Asp was administered for 15 days followed by MPs for an additional 30 days (D-Asp/PS-MP group). Finally, in an additional group, D-Asp was subsequently administered for 15 days to PS-MP-treated rats for 30 days (PS-MP/D-Asp group) to understand whether the amino acid exerts an alleviating role in the damage induced by PS-MPs on testicular activity.

As the treatment of the D-Asp/PS-MP and PS-MP/D-Asp groups lasted for 45 days, we decided to include a second control group comprising rats housed for 45 days. However, as we found no differences between the two control groups (C 30 and C 45; for details, see the Materials and Methods) for all parameters considered, we used the mean values observed from both C groups for all graphical representations of biochemical and morphological analyses. Therefore, from here on, we will refer to both as controls (C).

### 3.1. Histology

**Figure 1:**
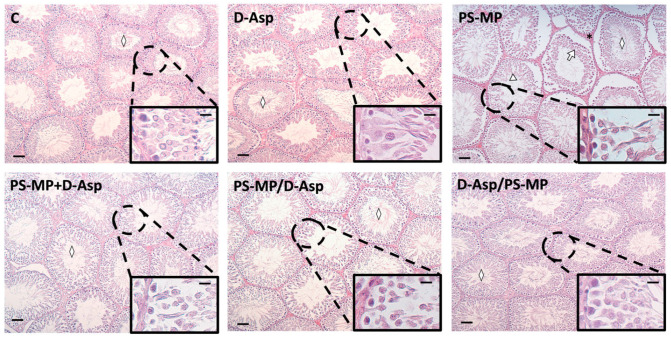
Hematoxylin–eosin staining of paraffin-embedded rat testicular sections from C and D-Asp, PS-MPs, PS-MPs + D-Asp, PS-MPs/D-Asp-, and D-Asp/PS-MPs-treated groups. The images were captured at ×10 magnification (scale bars = 40 µm) and ×40 (scale bars = 10 µm) for the insets. Rhombus, tubular lumen; arrows, GC desquamation; triangles, spaces between GC.

Figure 1 shows representative histological images of testicular sections. In both the C and D-Asp-treated groups, the testes exhibited a typical organization of germinal and interstitial compartments. GCs were present at all stages of differentiation, with mature SPZ filling the tubular lumina (rhombuse), and LC and normal blood vessels in the interstitium. In contrast, testes from PS-MPs-treated rats displayed abnormal seminiferous tubules characterized by overall tissue disorganization. This was evident through disrupted connections between GC and the presence of empty spaces between them (triangle). Additional alterations included desquamation of GC from the basal membrane (arrow), hemorrhage, and dilated blood vessels in the interstitial space (asterisks). Notably, in the PS-MP + D-Asp, PS-MP/D-Asp, and D-Asp/PS-MP groups, the histological architecture—including the seminiferous epithelium and interstitial compartments—closely resembled that of the C testes.

As shown in Table 1, the analysis of three key morphometric parameters further corroborated the histological findings. Specifically, the tubular diameter, epithelium thickness, and percentage of tubule lumens filled with SPZ were all significantly reduced in the PS-MP-treated group compared to both the C (*p* < 0.01 for all parameters).

Treatment with D-Asp partially alleviated the detrimental effects of PS-MP exposure. In the PS-MP + D-Asp, PS-MP/D-Asp, and D-Asp/PS-MP groups, all three parameters showed significant improvement compared to the PS-MP group (*p* < 0.01). However, values in these groups remained significantly lower than those of the C group (*p* < 0.01), except for epithelium thickness in the D-Asp/PS-MP group, which was comparable to C values.

### 3.2. Oxidative Stress

**Figure 2:**
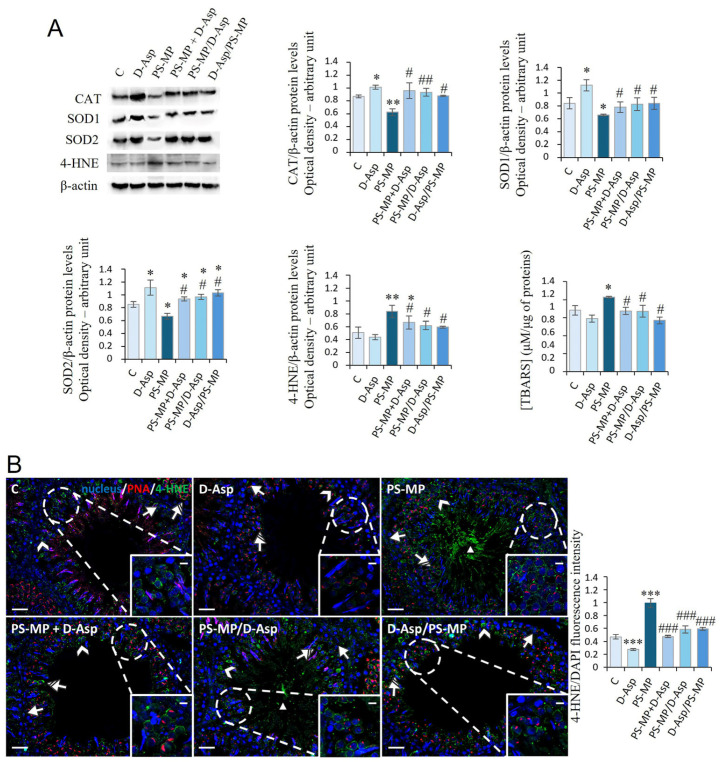
Evaluation of oxidative stress in C and D-Asp, PS-MPs, PS-MPs + D-Asp-, PS-MPs/D-Asp, and D-Asp/PS-MPs-treated rat testis. (**A**) WB analysis of CAT (60 kDa), SOD1 (26 kDa), SOD2 (23 kDa), 4-HNE (66 kDa), and TBARS assay in the testes of rats treated with D-Asp and/or PS-MPs. The amount of CAT, SOD1, SOD2, and 4-HNE was quantified using ImageJ program and normalized with respect to β-actin (42 kDa). (**B**) Testicular 4-HNE (green) immunolocalization. The slides were counterstained with PNA (red) and DAPI-fluorescent nuclear staining (blue). The images were captured at ×20 magnification (scale bars = 20 µm) and ×40 (scale bars = 10 µm) for the insets. Arrows, SPG; arrowheads, SPC; striped arrows, SPT; triangle, SPZ tails. The histogram shows the quantification of 4-HNE fluorescence signal intensity. All values are expressed as means ± SD from five animals in each group. * *p* < 0.05; ** *p* < 0.01; *** *p* < 0.001, D-Asp, PS-MP, PS-MP + D-Asp-, PS-MP/D-Asp- and D-Asp/PS-MP vs. C. # *p* < 0.05; ## *p* < 0.01; ### *p* < 0.001, PS-MP + D-Asp-, PS-MP/D-Asp-, and D-Asp/PS-MP vs. PS-MP.

Figure 2A shows the expression levels of the antioxidant enzymes, CAT, SOD1, and SOD2 after PS-MPs and/or D-Asp exposure. PS-MPs induced a significant reduction in all three enzymes when compared with C (CAT, *p* < 0.01; SOD1, SOD2, *p* < 0.05), while D-Asp produced an increase in its expression (*p* < 0.05). In all three groups (PS-MP + D-Asp, PS-MP/D-Asp, and D-Asp/PS-MP), the protein levels of the antioxidant enzymes significantly increased (*p* < 0.01–0.05) compared to the PS-MP group, reaching levels similar to or higher than C (Figure 2A). 4-HNE is an α,β-unsaturated hydroxyalkenal produced by lipid peroxidation, which can form adducts with proteins. PS-MPs induced a significant increase in 4-HNE protein levels when compared with C (*p* < 0.01); in the D-Asp-treated group, 4-HNE levels were unchanged. In all three groups (PS-MP + D-Asp, PS-MP/D-Asp, and D-Asp/PS-MP), 4-HNE expression resulted in a significant reduction (*p* < 0.05) compared to the PS-MP group (Figure 2A).

IF analysis of 4-HNE (Figure 2B) revealed scattered 4-HNE-positive cells in the C, D-Asp, PS-MP + D-Asp, and D-Asp/PS-MP testicular sections. Positive signals were mainly observed in spermatogonia (SPG; arrow), spermatocytes (SPC; arrowhead and inset), and spermatids (SPT; striped arrow). In addition, testes from the PS-MP and PS-MP/D-Asp groups showed 4-HNE positivity in the tails of luminal spermatozoa (SPZ; triangle). Quantitative analysis of fluorescence intensity supported the WB findings, showing a significant increase in 4-HNE signal in the PS-MP group compared to C (*p* < 0.001; Figure 2B). Notably, 4-HNE fluorescence was significantly reduced in all D-Asp-treated groups (PS-MP + D-Asp, PS-MP/D-Asp, and D-Asp/PS-MP) compared to the PS-MP group (*p* < 0.001). Interestingly, D-Asp treatment alone led to a significant reduction in 4-HNE signal intensity compared to the C (*p* < 0.001), suggesting a potential antioxidant effect of D-Asp even under physiological conditions.

The oxidative stress induced by PS-MP was also analyzed by measuring the testicular levels of TBARS, another byproduct of lipid peroxidation. PS-MP increased the levels of TBARS (*p* < 0.05) compared to those of C. In PS-MP + D-Asp, PS-MP/D-Asp, and D-Asp/PS-MP groups, the levels of TBARS were significantly lower (*p* < 0.05) than the PS-MP group and almost comparable to those of C (Figure 2A).

### 3.3. Apoptosis

**Figure 3:**
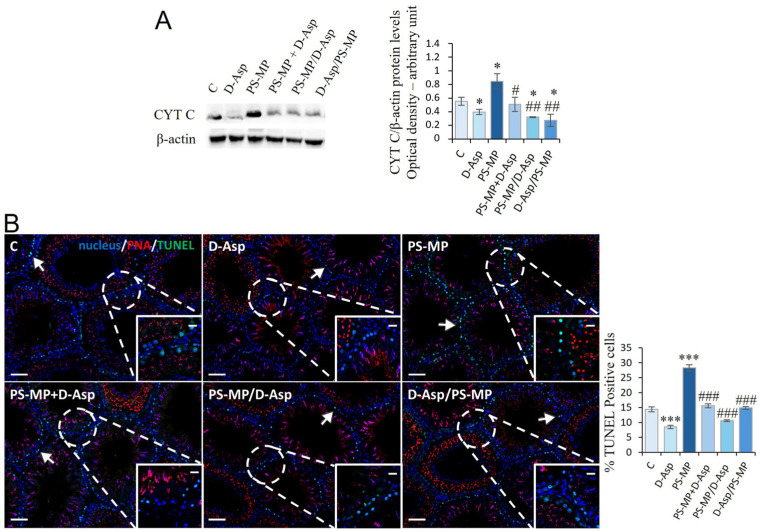
Apoptotic rate of C and D-Asp, PS-MPs, PS-MPs + D-Asp-, PS-MPs/D-Asp-, and D-Asp/PS-MPs-treated rat testis. (**A**) WB analysis of CYT C (14 kDa) protein levels in the testis of animals treated with D-Asp and/or PS-MP. The amount of CYT C was quantified using ImageJ program and normalized with respect to β-actin (42 kDa). (**B**) Determination of apoptotic cells through the detection of TUNEL-positive cells (green). The slides were counterstained with PNA lectin (red) and with DAPI-fluorescent nuclear staining (blue). The images were captured at ×10 magnification (scale bars = 50 µm) and ×20 (scale bars = 20 µm) for the insets. Arrows, SPG. The histogram shows the percentage of TUNEL-positive cells. All the values are expressed as means ± SD from five animals in each group. * *p* < 0.05; *** *p* < 0.001, D-Asp, PS-MP, PS-MP + D-Asp-, PS-MP/D-Asp- and D-Asp/PS-MP vs. C. # *p* < 0.05; ## *p* < 0.01; ### *p* < 0.001, PS-MP + D-Asp-, PS-MP/D-Asp- and D-Asp/PS-MP vs. PS-MP.

The expression levels of CYT C were examined to evaluate the effects of PS-MPs and/or D-Asp administration on apoptosis in the rat testis (Figure 3A). WB analysis showed that D-Asp treatment provoked a slight decrease in CYT C expression levels (*p* < 0.05), whereas PS-MPs treatment induced a significant increase in its expression level (*p* < 0.05) compared to C (Figure 3A). In the PS-MP + D-Asp group, CYT C levels were lower than the PS-MP group (*p* < 0.05) and reached the C value (Figure 3A). D-Asp given at different times to PS-MPs (PS-MP/D-Asp and D-Asp/PS-MP) reduced CYT C expression, which resulted in significantly lower levels with respect to the PS-MP group (*p* < 0.01) and C group (*p* < 0.05) (Figure 3A).

To further support these findings, a TUNEL assay was performed (Figure 3B). The results revealed the presence of dispersed apoptotic cells in both the C and D-Asp-treated groups, primarily among SPG (arrows) (Figure 3B). Interestingly, D-Asp treatment alone led to a 41% reduction in the number of apoptotic cells compared to C (*p* < 0.001; Figure 3B), indicating a potential anti-apoptotic effect under normal conditions. In contrast, exposure to PS-MP resulted in a 97% increase in TUNEL-positive cells, particularly SPG, compared to C (*p* < 0.001), supporting its pro-apoptotic impact. Co-treatment with D-Asp markedly attenuated PS-MPs-induced apoptosis. Specifically, when D-Asp was administered together with, before, or after PS-MPs exposure, the number of TUNEL-positive cells decreased by 28%, 62%, and 47%, respectively, relative to the PS-MP group (*p* < 0.001 in all cases).

### 3.4. Autophagy

**Figure 4:**
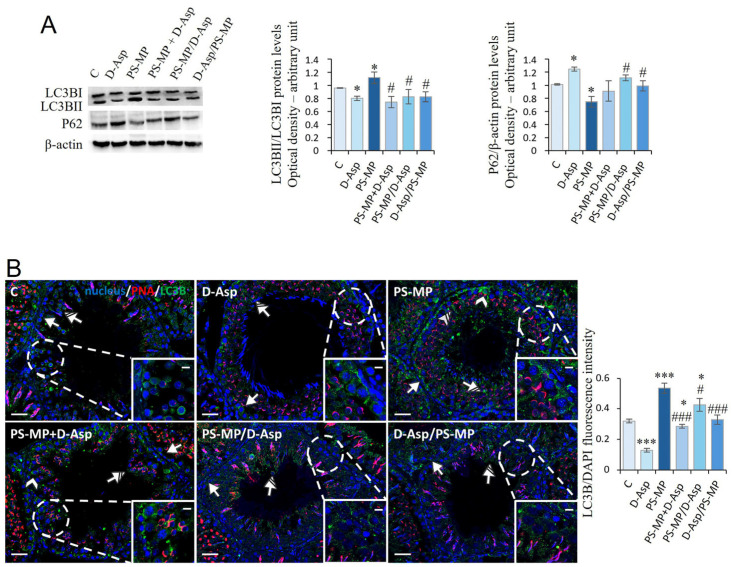
Autophagy rate analysis of C and D-Asp-, PS-MPs-, PS-MPs + D-Asp-, PS-MPs/D-Asp-, and D-Asp/PS-MPs-treated rat testis. (**A**) WB analysis of LC3BI (14 kDa), LC3BII (16 kDa), and P62 (62 kDa) protein levels in the testis of animals treated with D-Asp and/or PS-MP. Histograms show the LC3BII/LC3BI ratio and P62 relative protein levels, respectively. Protein levels were quantified using the ImageJ program and normalized versus β-actin (42 kDa). (**B**) Testicular LC3B (green) immunolocalization. The slides were counterstained with PNA (red) and DAPI-fluorescent nuclear staining (blue). The images were captured at ×20 magnification (scale bars = 20 µm) and ×40 (scale bars = 10 µm) for the insets. Arrows, SPG; arrowheads, SPC; striped arrows, SPT; striped arrowheads, SC. The histogram shows the quantification of LC3B fluorescence signal intensity. All values are expressed as means ±SD from five animals in each group. * *p* < 0.05; *** *p* < 0.001, D-Asp, PS-MP, PS-MP + D-Asp-, PS-MP/D-Asp- and D-Asp/PS-MP vs. C. # *p* < 0.05; ### *p* < 0.001, PS-MP + D-Asp-, PS-MP/D-Asp-, and D-Asp/PS-MP vs. PS-MP.

In rat testis, the autophagy process was analyzed via the expression of LC3BI, LC3BII, and p62 proteins (Figure 4A). We found that the ratio of LC3BII/LC3BI was significantly higher (*p* < 0.05) in PS-MPs-treated rats compared to that of C, whereas the p62 protein levels significantly decreased (*p* < 0.05) (Figure 4A). Vice versa, in the D-Asp-treated group, LC3BII/LC3BI and p62 protein levels were significantly (*p* < 0.05) lower and higher than C, respectively (Figure 4A). In PS-MP + D-Asp PS-MP/D-Asp and D-Asp/PS-MP groups, LC3BII/LC3BI demonstrated results significantly (*p* < 0.05) lower than the PS-MP group, whereas p62 protein levels were higher (Figure 4A).

To identify the cell types most actively involved in autophagy, LC3B IF staining was performed (Figure 4B). Across all experimental groups, positive LC3B signals were detected in the cytoplasm of GC, particularly SPG (arrow) and SPT (striped arrow). In the PS-MPs-treated group, additional LC3B expression was observed in SPC (arrowhead) and in the cytoplasmic extensions of SC (striped arrow and inset), indicating broader activation of the autophagic response. Quantitative analysis of fluorescence intensity showed that LC3B expression was significantly reduced in the D-Asp-treated group (*p* < 0.001) and markedly increased in the PS-MP group (*p* < 0.001) compared to C, suggesting a suppressive effect of D-Asp and an autophagy-inducing effect of PS-MP exposure.

In the PS-MP + D-Asp (*p* < 0.001), PS-MP/D-Asp (*p* < 0.05), and D-Asp/PS-MP (*p* < 0.001) groups, LC3B fluorescence intensity was significantly lower than in the PS-MP group, indicating that D-Asp administration mitigated PS-MPs-induced autophagy activation. Notably, only in the D-Asp/PS-MP group, where D-Asp was administered as a preventive treatment, LC3B signal intensity was comparable to that of C.

### 3.5. Steroidogenesis

Table 2 reports testis T levels, which showed a significant increase in D-Asp-treated rats (*p* < 0.01) and a significant decrease in PS-MP-treated animals (*p* < 0.001) compared to those of C. In the PS-MP + D-Asp, PS-MP/D-Asp, and D-Asp/PS-MP-treated groups, T levels were significantly higher than PS-MP group (*p* < 0.001–0.01). However, only the PS-MP + D-Asp T levels reached C values.

**Figure 5:**
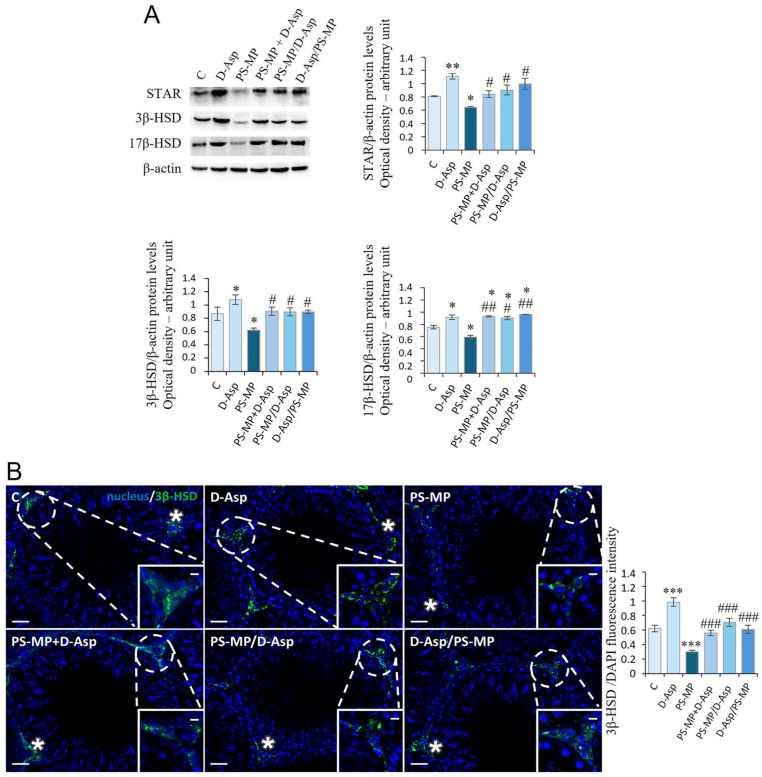
Steroidogenesis analysis of C and D-Asp-, PS-MP-, PS-MP + D-Asp-, PS-MP/D-Asp-, and D-Asp/PS-MP-treated rat testis. (**A**) WB analysis of StAR (32 kDa), 3β-HSD (42 kDa), and 17β-HSD (35 kDa) protein levels in the testis of animals treated with D-Asp and/or PS-MP. The amount of StAR, 3β-HSD, and 17β-HSD was quantified using ImageJ program and normalized with respect to β-actin (42 kDa). (**B**) Testicular 3β-HSD (green) immunolocalization. The slides were counterstained with DAPI-fluorescent nuclear staining (blue). The images were captured at ×20 magnification (scale bars = 20 µm) and ×40 (scale bars = 10 µm) for the insets. Asterisks, LC. The histogram shows the quantification of 3β-HSD fluorescence signal intensity. All values are expressed as means ± SD from five animals in each group. * *p* < 0.05; ** *p* < 0.01; *** *p* < 0.001, D-Asp, PS-MP, PS-MP + D-Asp-, PS-MP/D-Asp- and D-Asp/PS-MP vs. C. # *p* < 0.05; ## *p* < 0.01; ### *p* < 0.001, PS-MP + D-Asp-, PS-MP/D-Asp-, and D-Asp/PS-MP vs. PS-MP.

We evaluated the expression levels of StAR and steroidogenic enzymes (3β-HSD, 17β-HSD) in the testes of experimental groups and C (Figure 5A). WB analysis showed that D-Asp treatment provoked an increase in StAR (*p* < 0.01), 3β-HSD (*p* < 0.05), and 17β-HSD (*p* < 0.05) protein levels, while PS-MP treatment induced a significant decrease in their expression (*p* < 0.05) compared to C (Figure 5A). D-Asp, either administered at the same time with MPs or at different times (PS-MP + D-Asp, PS-MP/D-Asp, and D-Asp/PS-MP), increased StAR, 3β-HSD, and 17β-HSD protein levels (*p* < 0.01–0.05) compared to the PS-MP group; 17β-HSD protein levels were higher (*p* < 0.05) when compared to C values (Figure 5A).

The effects of D-Asp and PS-MP on steroidogenesis were further evaluated by IF staining for 3β-HSD, as shown in Figure 5B. In all experimental groups, the 3β-HSD signal was exclusively localized in interstitial LC (asterisks), confirming its cell-specific expression. Quantitative analysis of fluorescence intensity revealed a significantly stronger signal in the D-Asp-treated group compared to C (*p* < 0.001), indicating enhanced steroidogenic activity. Conversely, the PS-MP-treated group exhibited a markedly reduced signal (*p* < 0.001), suggesting impaired steroidogenesis. Importantly, in the PS-MP + D-Asp, PS-MP/D-Asp, and D-Asp/PS-MP groups, 3β-HSD signal intensity was significantly higher than in the PS-MP group (*p* < 0.001) and comparable to C levels, indicating that D-Asp effectively mitigated PS-MPs-induced disruption of LC function.

### 3.6. Spermatogenesis

**Figure 6:**
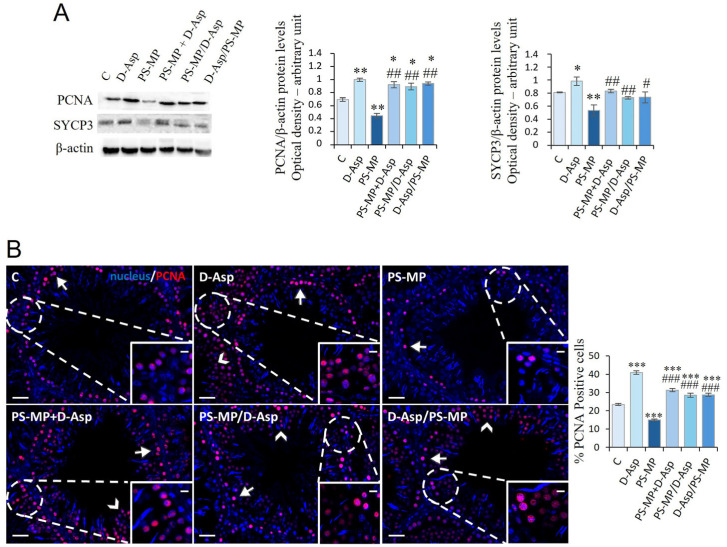
Spermatogenesis analysis of C and D-Asp-, PS-MP-, PS-MP + D-Asp-, PS-MP/D-Asp-, and D-Asp/PS-MP-treated rat testis. (**A**) WB analysis of PCNA (36 kDa) and SYCP3 (30–33 kDa) protein levels in the testis of animals treated with D-Asp and/or PS-MP. Protein levels were quantified using the ImageJ program and normalized versus β-actin (42 kDa). (**B**) Testicular PCNA (green) immunolocalization. The slides were counterstained with PNA (red) and DAPI-fluorescent nuclear staining (blue). The images were captured at ×20 magnification (scale bars = 20 µm) and ×40 (scale bars = 10 µm) for the insets. Arrows, SPG; arrowheads, SPC. The histogram shows the percentage of PCNA-positive cells. All the values are expressed as means ± SD from five animals in each group. * *p* < 0.05; ** *p* < 0.01; *** *p* < 0.001, D-Asp, PS-MP, PS-MP + D-Asp-, PS-MP/D-Asp-, and D-Asp/PS-MP vs. C. # *p* < 0.05; ## *p* < 0.01; ### *p* < 0.001, PS-MP + D-Asp-, PS-MP/D-Asp-, and D-Asp/PS-MP vs. PS-MP.

The spermatogenic activity in the rat testis was evaluated by the level expression of PCNA, a protein expressed in the S phase of the cell cycle, and SYCP3, a protein involved in forming the synaptonemal complex (Figure 6A). Treatment with D-Asp caused an increase in PCNA (*p* < 0.01) and SYCP3 (*p* < 0.05) protein levels, whereas treatment with PS-MPs induced its significant decrease (*p* < 0.01) in C (Figure 6A). In PS-MP + D-Asp, PS-MP/D-Asp, and D-Asp/PS-MP groups, PCNA and SYCP3 expression levels were significantly higher with respect to the PS-MP group (*p* < 0.05–0.01); the PCNA levels exceeded the values of C (*p* < 0.05) (Figure 6A).

PCNA immunolabeling was also performed to assess cell proliferation (Figure 6B). In all groups, PCNA-positive cells were primarily localized in the SPG layer at the basal compartment of the seminiferous tubules (arrows; Figure 6B). Interestingly, in the testes of animals treated with D-Asp, additional labeling was observed in SPC (arrowheads), as also shown in Figure 6B, suggesting a broader proliferative response. Quantitative analysis revealed that D-Asp treatment significantly increased the number of PCNA-positive cells by approximately 74% compared to C (*p* < 0.001), indicating enhanced cell cycle progression. Conversely, PS-MPs treatment significantly reduced PCNA expression by 36% compared to C (*p* < 0.001), indicating an inhibitory effect on GC proliferation. Notably, in all co-treatment and pre-/post-treatment groups (PS-MP + D-Asp, PS-MP/D-Asp, and D-Asp/PS-MP), the percentage of PCNA-positive cells was significantly higher than in both the PS-MPs-treated (*p* < 0.001) and C (*p* < 0.001), suggesting a robust stimulatory effect of D-Asp on spermatogenic cell proliferation, even under toxic conditions.

The effects of PS-MPs and/or D-Asp treatment were extended to gamete physiology. The analysis of the main sperm parameters, shown in Table 3, revealed that when given alone, PS-MPs induced substantial alterations in motility, viability, and morphology compared to C (*p* < 0.05–0.01). D-Asp, simultaneously or at different times administered with PS-MPs, counteracted the dangerous PS-MP-induced effects on sperm parameters.

## 4. Discussion

Among MPs, PS-MPs are more widespread pollutants in terrestrial and marine ecosystems [13,14], and they are extensively studied for their toxicological effects on reproduction [1,3,45,46,47]. PS-MPs can enter and accumulate in testicular tissue through blood circulation, destroying the BTB and impairing testicular activity [19,22,48,49]. Specifically, the harmful effects they cause in the testis include increased oxidative stress, autophagy, and apoptosis; a reduction in T biosynthesis; disruption of the cytoskeleton of somatic cells and GCs; and ultimately, impairment of sperm quality [18,19,23,24].

Given the widespread exposure to MPs and the magnitude of damage caused by them, there is an increasing demand for natural dietary molecules able to intervene and alleviate MPs’ toxicity. In this study, we evaluated, for the first time, the potential role of D-Asp in alleviating the PS-MPs-induced damage on the rat testis. The experimental design consisted of D-Asp oral treatment, performed contemporaneously or given at different times with PS-MPs, to adult rats. Specifically, D-Asp was administered simultaneously to PS-MPs to investigate its role in counteracting the testicular damage caused by PS-MPs. In addition, D-Asp was administered previously to PS-MPs or subsequently to PS-MPs to understand whether the amino acid exerted a preventive or alleviating role in the damage induced by PS-MPs on testicular activity, respectively.

D-Asp, an amino acid endogenously present in vertebrate testicular parenchyma, may be produced by the degradation of proteins derived from the diet or intestinal bacteria containing the amino acid [32]. The enzyme aspartate racemase, which catalyzes the synthesis of D-Asp by L-Aspartate, contributes to the tissue homeostasis of the amino acid [50]. Studies carried out in animal models have shown that D-Asp, administered orally or intraperitoneally, accumulates in the testes and modulates testicular activity [34]. D-Asp exhibits various biochemical and pharmacological properties, including antioxidant and anti-apoptotic effects [28,29,51]. The amino acid promotes spermatogenesis, not only by activating T biosynthesis in LC but also by triggering autocrine and/or paracrine signaling in testicular cells, which are mediated by the GluR/ERK pathway [36,50,52,53,54,55]. Further, D-Asp, by increasing the expression of PREP and DAAM, two proteins involved in cytoskeleton remodeling, promotes gamete differentiation [52,56]. Finally, studies in humans demonstrated that D-Asp levels in semen of males affected by oligospermia or azoospermia were lower compared to those of normospermics, strongly supporting the key role played by the amino acid in male fertility [38].

Despite the increasing interest in D-Asp for its reproductive benefits, no previous in vivo studies have investigated its potential to mitigate testicular damage specifically caused by PS-MPs. The present work addresses this gap, providing the first experimental evidence of D-Asp’s protective action against PS-MP-induced testicular dysfunction.

According to data present in [19,23,48,57], our results confirmed that exposure to PS-MPs induces histological disorganization in testicular parenchyma, indicated by disrupted connections between GC, desquamation of GC from the basal membrane, dilated blood vessels in the interstitial space, and reduced percentage of tubule lumens filled with SPZ. Treatment with D-Asp alleviated the detrimental effects of PS-MP exposure, as well as if the histological parameters did not reach those of C.

Furthermore, our results confirmed that exposure to PS-MPs induces an increase in oxidative stress in rat testis, suggested by the decreased expression levels of the antioxidant enzymes (SOD and CAT) and the increased expression levels of 4-HNE and concentration of TBARS, indicative of augmented lipid peroxidation. Quantitative analysis of fluorescence intensity of 4-HNE supported the WB findings, showing a significant intensification in 4-HNE signal in the PS-MP group, particularly in SPG, and a significant reduction in all D-Asp-treated groups (PS-MP + D-Asp, PS-MP/D-Asp, and D-Asp/PS-MP) compared to the PS-MP group, suggesting a potential antioxidant effect of D-Asp even under toxic conditions. In line with these findings, some studies suggested that D-Asp may alleviate the oxidative stress induced by environmental pollutants in the testis by affecting the SOD and CAT activities and the TBARS levels [29,42].

As a consequence of the establishment of an oxidative stress state, we observed enhanced apoptosis in PS-MPs-treated rat testis, evidenced by upregulated expression of CYT C as well as an increased percentage (97%) of TUNEL-positive cells, highlighting the pro-apoptotic impact of the PS-MPs, particularly in SPG. Apoptosis refers to the process of programmed cell death to maintain the stability of the testicular environment. Numerous studies report that MPs can induce apoptosis in various tissues, including the testis [17,19,23,58,59]. Notably, when D-Asp was administered together with, before, or after PS-MP exposure, the amino acid restored the initial values of antioxidant enzyme expression, showing its ability to counteract the adverse effects induced by PS-MPs. Biochemical results were strongly supported by the decreased number (28–62%) of TUNEL-positive cells relative to the PS-MP group. We hypothesized that the antioxidant action exerted by D-Asp reduced the apoptotic process, maintaining a normal testicular environment.

Excessive oxidative stress can activate the autophagic pathway, an adaptive mechanism that leads to the lysosomal degradation of damaged molecules and organelles. Our results, according to a previous study [18], showed that PS-MPs stimulate autophagy, as suggested by increased expression of LC3BI and LC3BII and decreased p62 proteins. IF analysis showed positive LC3B signals in SPG, SPC, SPT, and SC. The localization patterns of 4-HNE (oxidative stress marker) and LC3B (autophagy marker) provide valuable insights into the cellular responses to oxidative stress within the testicular environment. In GC, the expression of both 4-HNE and LC3B suggests that oxidative stress may directly trigger autophagic processes, likely as a protective or adaptive response. In contrast, SC exhibited LC3B positivity in the apparent absence of 4-HNE staining, indicating that autophagy in these cells could not be driven by oxidative stress. This observation points instead to a possible involvement of autophagy in compensatory mechanisms associated with functional impairments of the BTB or disrupted junctional complexes with GC. Notably, exposure to environmental toxicants, including MPs, has been shown to compromise BTB integrity [16,60,61]. Further studies are needed to explore the specific impact of PS-MPs on the BTB and autophagic processes in SC, with the aim of better understanding the molecular mechanisms underlying these disruptions and their potential long-term effects on testicular health. In addition, here, we demonstrated, for the first time, that treatment with D-Asp alone induces a significant reduction in the expression of LC3BI and LC3BII and an increase in p62 proteins, indicating anti-autophagic activity of the amino acid in physiological conditions. However, more importantly, the amino acid showed the ability to counteract/prevent the increase in this process induced by PS-MPs.

We further confirmed that PS-MPs disturb testicular steroidogenesis [16,19,24,62,63], as our results showed that in the testis of PS-MPs-treated rats, the expression levels of StAR, 3β-HSD, and 17β-HSD decreased, resulting in reduced testicular T. Previous reports demonstrated that PS-MPs reduce serum T levels by repressing the expression of steroidogenic genes and proteins in mice and rats [19,24]. Reduced protein levels of StAR, 3β-HSD, and 17β-HSD were also detected in mouse Leydig TM3 cells exposed to PS-MPs [26]. We found that in the experimental groups in which D-Asp was administered simultaneously or at different times, the amino acid counteracted the impaired effects induced by PS-MPs on steroidogenesis, as evidenced by elevated testicular T levels and enhanced expression of steroidogenic markers. These results are strongly supported by IF positivity for 3β-HSD, exclusively localized in interstitial LC, confirming its cell-specific expression.

Considering that T regulates the whole spermatogenetic process, its decreased level may be one of the main factors responsible for the impaired spermatogenesis in PS-MPs-treated rats. Indeed, we found that PS-MPs reduced the expression levels of PCNA and SYCP3, markers of GC proliferation, and altered the sperm parameters, such as motility, viability, and morphology. In this regard, Hassine et al. (2023) [18] hypothesized that one of the causes of altered sperm motility may be due to the downregulation of PREP protein levels induced by PS-MPs. PREP is a serine protease localized in the sperm tail, implicated in microtubule-associated processes [64]. Notably, our previous study demonstrated that the oral administration of D-Asp to adult rats upregulates PREP protein expression in the testis [50]. In this regard, we found that D-Asp, administered simultaneously or at different times, counteracted the impaired effects induced by PS-MPs, as evidenced by restored PCNA and SYCP3 expression levels and normalized sperm parameters. Interestingly, IF analysis revealed that in C and PS-MP groups, PCNA positivity was prevalently localized in the SPG, whereas in all D-Asp-treated groups, additional labeling was observed in SPC, suggesting a robust stimulatory effect of D-Asp on GC proliferation, even under toxic conditions. Accordingly, in vivo and in vitro studies have widely demonstrated that D-Asp participates in GC proliferation by enhancing PCNA, mitosis (Aurora B, pH3), and meiosis (Sycp3) markers in mouse and rat testes [28,29,35,36,37,56]. Consistently, Ddo knock-in mice, which exhibit low testicular D-Asp concentrations, showed lower levels of PCNA and SYCP3 compared to wild-type mice [65]. In addition, previous in vivo studies from our group demonstrated that D-Asp exerts marked protective effects against different testicular toxicants, including Cd and EDS [29,42,66]. Although all these agents compromise testicular function mainly through disruption of steroidogenesis, oxidative stress, and apoptosis, D-Asp mitigates their effects via partially overlapping yet toxin-specific mechanisms. In Cd-exposed rats, D-Asp preserves mitochondrial integrity, rebalances fusion–fission dynamics, and restores biogenesis and mitophagy while re-establishing ER-mitochondria crosstalk and lipid transfer essential for steroidogenesis [29,66]. Conversely, in the EDS model, where LC ablation causes T depletion and secondary GC apoptosis, D-Asp primarily safeguards LC survival and steroidogenic activity, possibly through a transesterification reaction that neutralizes the alkylating potential of EDS, together with the reinforcement of antioxidant defenses. These dual protective actions converge in maintaining hormonal homeostasis, reducing GC death, and promoting spermatogenic recovery [42].

## 5. Conclusions

This study is the first to provide in vivo evidence of the protective action of D-Asp against testicular dysfunction induced by PS-MPs. D-Asp administration effectively attenuated PS-MPs-induced oxidative stress, apoptosis, and autophagy and significantly restored testicular architecture and function. Furthermore, the amino acid enhanced the expression of key steroidogenic and spermatogenic markers, leading to the recovery of T levels and improvement in sperm parameters. Notably, D-Asp exerted its beneficial effects regardless of the timing of administration relative to PS-MP exposure, suggesting both preventive and therapeutic potential. These findings not only expand the current understanding of the toxicological impact of PS-MPs on male reproductive health but also highlight D-Asp as a promising natural compound with translational relevance for the development of protective strategies against environmental reprotoxicity. However, translating these findings into a practical and safe intervention for humans requires further studies to bridge the gap between animal and human dosages.

## Figures and Tables

**Table 1:** Morphometric parameters in control (C) and D-Asp- and/or PS-MP-treated rat testis.

	Tubules Diameter (µm)	Epithelium Thickness (µm)	Empty Lumen (%)
**C**	241.9 ± 4.8	52.3 ± 4.7	42.1 ± 2.4
**D-Asp**	247.1 ± 5.9	55.6 ± 4.5	38.8 ± 5.9
**PS-MP**	186.0 ± 7.6 **	40.7 ± 9.1 **	61.6 ± 4.5 **
**PS-MP + D-Asp**	214.1 ± 5.1 **^, ##^	48.3 ± 3.3 **^, ##^	50.7 ± 6.6 **^, ##^
**PS-MP/D-Asp**	210.5 ± 9.8 **^, ##^	44.4 ± 6.4 **^, #^	49.1 ± 3.2 **^, ##^
**D-Asp/PS-MP**	224.3 ± 6.5 **^, ##^	50.6 ± 3.8 **^, ##^	46.5 ± 6.6 **^, ##^

All values are expressed as means ± SD from five animals for group. ^#^, *p* < 0.05; **, ^##^, *p* < 0.01. *, vs. C; ^#^, vs. PS-MP.

**Table 2:** Testosterone levels in control (C) and D-Asp- and/or PS-MP-treated rat testis.

	ng/g Tissue
**C**	18.3 ± 2.2
**D-Asp**	28.8 ± 3.4 **
**PS-MP**	2.06 ± 0.6 ***
**PS-MP + D-Asp**	17.56 ± 1.7 ^###^
**PS-MP/D-Asp**	9.06 ± 0.9 **^, ##^
**D-Asp/PS-MP**	13.25 ± 1.1 *^, ##^

All values are expressed as means ± SD from five animals for each group. *, *p* < 0.05; **, ^##^, *p* < 0.01; ***, ^###^, *p* < 0.001. *, vs. C; #, vs. PS-MP.

**Table 3:** Sperm parameters in control (C) and D-Asp- and/or PS-MP-treated rat testis.

	Motility (%)	Viability (%)	Abnormal Morphology (%)
**C**	85.2 ± 7.3	98.8 ± 0.4	7.8 ± 1.7
**D-Asp**	92.2 ± 4.1	97.6 ± 0.6	6.2 ± 1.1
**PS-MP**	45.9 ± 3.6 *	78.3 ± 2.3 *	31.4 ± 6.8 **
**PS-MP + D-Asp**	81.3 ± 12.2 ^#^	93.4 ± 2.3 ^#^	21.2 ± 3.9 *
**PS-MP/D-Asp**	85.7 ± 8.1 ^##^	96.7 ± 1.6 ^#^	12.6 ± 0.2 ^#^
**D-Asp/PS-MP**	68.4 ± 3.1 ^#^	97.1 ± 1.3 ^#^	13.2 ± 1.7 ^#^

All values are expressed as means ± SD from five animals for each group. *, ^#^, *p* < 0.05; **, ^##^, *p* < 0.01; *, vs. C; ^#^, vs. PS-MP.

## Data Availability

The original contributions presented in this study are included in the article/Supplementary Material. Further inquiries can be directed to the corresponding authors.

## Supplementary Materials

The following supporting information can be downloaded at: https://www.mdpi.com/article/10.3390/biom15111484/s1, Table S1: The primary antibodies used in the Western blot analysis. Original WB image.

## Abbreviations

The following abbreviations are used in this manuscript:

17β-HSD	17β-Hydroxysteroid dehydrogenase
3β-HSD	3β-Hydroxysteroid dehydrogenase
4-HNE	4-Hydroxynonenal
BTB	Blood-testis barrier
CAT	Catalase
CYT C	Cytochrome c
DAAM1	Dishevelled-associated activator of morphogenesis 1
DAPI	4′,6-diamidino-2-phenylindole
D-Asp	D-Aspartate
GC	Germ cells
IF	Immunofluorescence
LC	Leydig cells
LC3B	Microtubule-associated proteins 1A/1B light chain 3B
MPs	Microplastics
p62	Sequestosome 1
PCNA	Proliferating Cell Nuclear Antigen
PNA	Peanut agglutinin
PREP	Prolyl endopeptidase
PS-MPs	Polystyrene microplastics
SC	Sertoli cells
SD	standard deviation
SOD1	Superoxide dismutase 1
SOD2	Superoxide dismutase 2
SPC	Spermatocytes
SPG	Spermatogonia
SPT	Spermatids
SPZ	Spermatozoa
StAR	Steroidogenic acute regulatory protein
SYCP3	Synaptonemal complex protein 3
T	Testosterone
TBARS	Thiobarbituric acid reactive substances
TUNEL	Terminal Transferase dUTP Nick End Labeling
WB	Western Blot
