# Supplementary material for: Protective Effects and Potential Mechanisms of D-Aspartate on Testicular Damage Induced by Polystyrene Microplastics
Source: Biomolecules. 2025 Oct 22;15(11):1484. doi: 10.3390/biom15111484 (PMC12650146; doi:10.3390/biom15111484)
Supplement: Supplementary file 1 [file biomolecules-15-01484-s001.zip › biomolecules-3890382 Supplementary original WB images.pdf]

IMAGES OF ELECTROPHORETIC BLOTS

The bands in the red box are those shown in the relative figure and they refer to the following samples: C 15; D-Asp; PS-MP; PS-MP + D-Asp; PS-MP/D-Asp; D-Asp/PS-MP; C 45.
We used the mean values observed from both C groups for all graphical representations of analyses.
Therefore, we will refer to both as controls (C) and we showed only one of the two bands (C 15 specifically) in the blots.

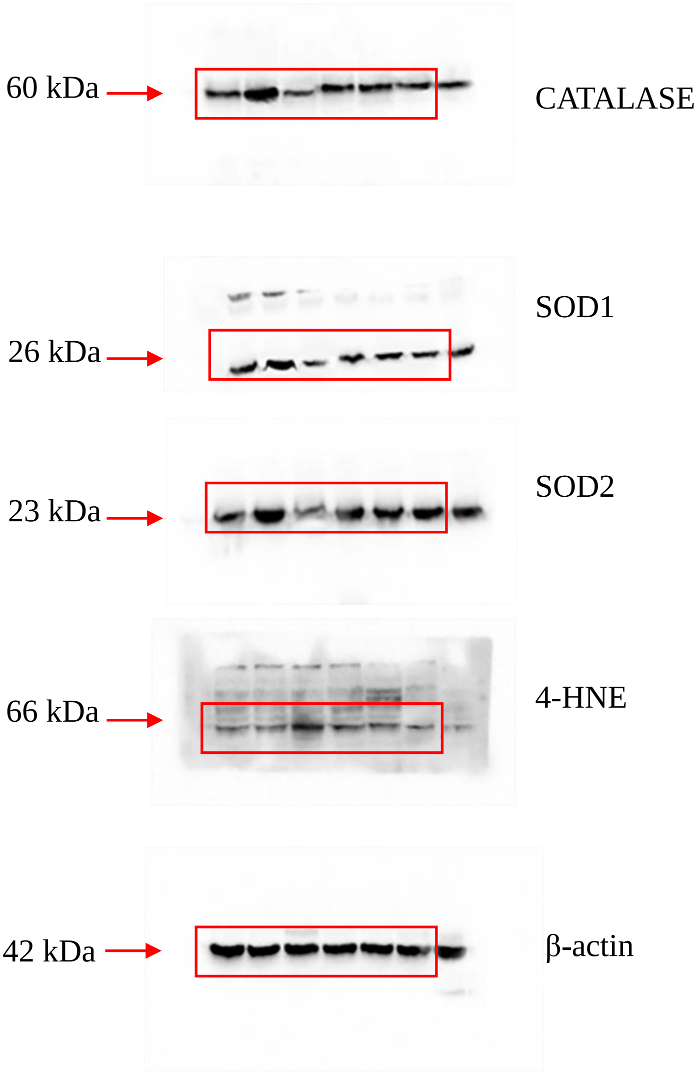

Figure 2

IMAGES OF ELECTROPHORETIC BLOTS

The bands in the red box are those shown in the relative figure and they refer to the following samples: C 15; D-Asp; PS-MP; PS-MP + D-Asp; PS-MP/D-Asp; D-Asp/PS-MP; C 45.
We used the mean values observed from both C groups for all graphical representations of analyses.
Therefore, we will refer to both as controls (C) and we showed only one of the two bands (C 15 specifically) in the blots.

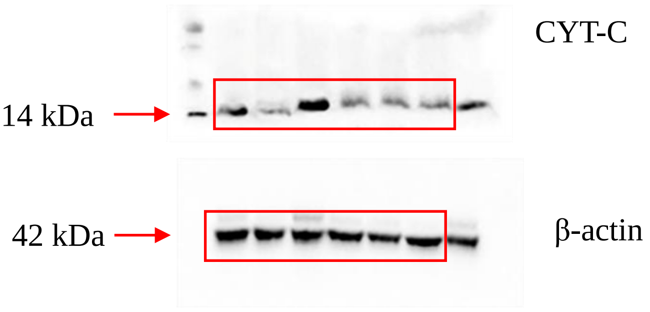

Figure 3

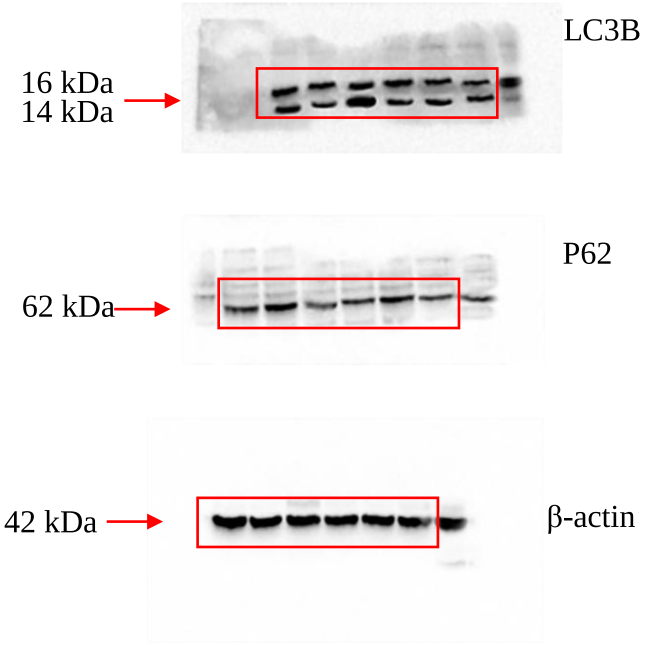

Figure 4

IMAGES OF ELECTROPHORETIC BLOTS

The bands in the red box are those shown in the relative figure and they refer to the following samples: C 15; D-Asp; PS-MP; PS-MP + D-Asp; PS-MP/D-Asp; D-Asp/PS-MP; C 45.
We used the mean values observed from both C groups for all graphical representations of analyses.
Therefore, we will refer to both as controls (C) and we showed only one of the two bands (C 15 specifically) in the blots.

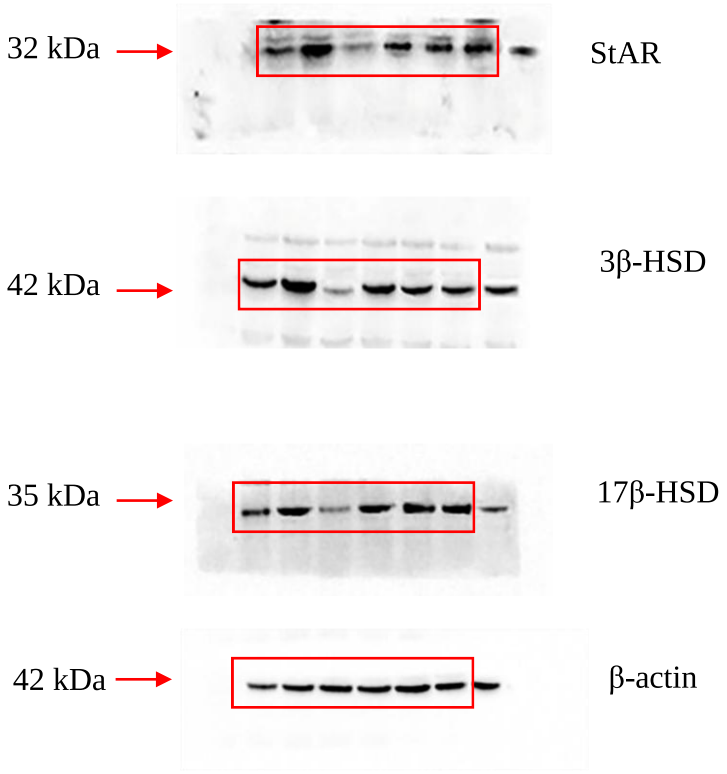

Figure 5

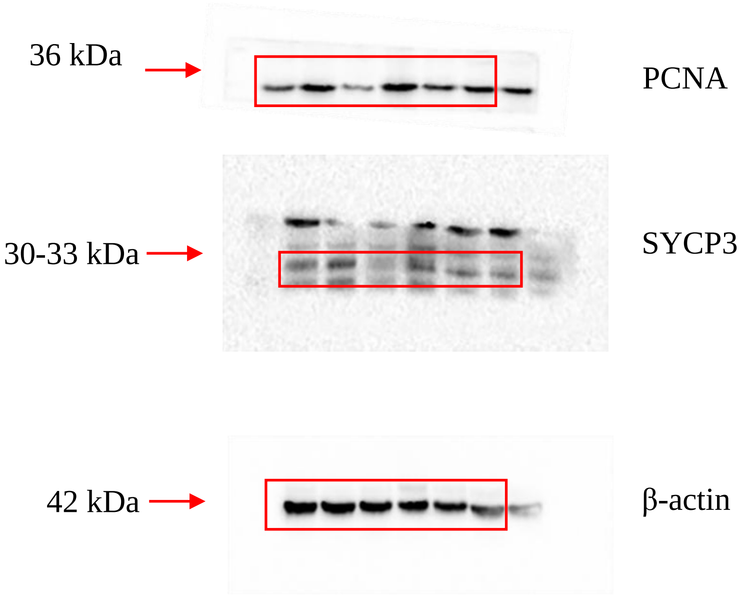

Figure 6
