# Supplementary material for: Protective Effects and Potential Mechanisms of D-Aspartate on Testicular Damage Induced by Polystyrene Microplastics
Source: Biomolecules. 2025 Oct 22;15(11):1484. doi: 10.3390/biom15111484 (PMC12650146; doi:10.3390/biom15111484)
Supplement: Supplementary file 1 [file biomolecules-15-01484-s001.zip › biomolecules-3890382-Supplementary Western blot analysis.pdf]

Table S1. The primary antibodies used in the Western blot analysis.

Antibody	Molecular weight (kDa)	WB Dilution	IF Dilution	Source
17β-HSD	35	1:1000	-	Santa Cruz Biotechnology, Santa Cruz, CA USA, #sc-32872
3β-HSD	42	1:1000	1:100	Elabscience Biotechnology, Wuhan, China #E-AB-15112
4-HNE	66	1:1000	1:100	Bioss Antibodies, Massachusetts, USA #BS-6313R
CAT	60	1:1000	-	Sigma-Aldrich, St. Louis, Missouri, USA #C0979
CYT C	14	1:1000	-	Cell Signaling Technology, Danvers, Ma, USA #4272
LC3B	15	1:1000	1:100	GeneTex, Inc., Irvine, California, USA #GTX82986
p-62	62	1:2000	-	Elabscience Biotechnology, Wuhan, China #E-AB-63539
PCNA	36	1:1000	1:100	Sigma-Aldrich, Milan, Italy #98825
SOD1	26	1:1000	-	Abcam, Cambridge, United Kingdom #ab13498
SOD2	22	1:2000	-	Abclonal, Massachusetts, USA #A1340
SYCP3	30-33	1:500	-	Santa Cruz Biotechnology, Santa Cruz, CA, USA #sc74569
StAR	32	1:1000	-	Elabscience Biotechnology, Wuhan, China #E-AB-15419
β-Actin	42	1:2000	-	Elabscience Biotechnology, Wuhan, China #E-AB-20031
Goat anti-rabbit IgG HRP	-	1:3000	-	Vector Laboratories, Burlingame, CA, USA #PI-1000
Goat anti-mouse IgG HRP	-	1:2000	-	BioActs, Namdong-gu, Incheon, Korea #RSA1122
Goat anti-rabbit Alexa Fluor 488	-	-	1:500	Thermo Fisher Scientific, Waltham, Ma, USA #A32731
Goat anti-mouse CF™ 568	-	-	1:500	Sigma-Aldrich, Saint Louis, MO, USA #SAB4600082
PNA Lectin Alexa Fluor 568	-	-	1:50	Thermo Fisher Scientific, Waltham, Ma, USA #L32458
